# Sleep loss impairs intestinal stem cell function and gut homeostasis through the modulation of the GABA signalling pathway in *Drosophila*

**DOI:** 10.1111/cpr.13437

**Published:** 2023-03-03

**Authors:** Juanyu Zhou, Li He, Mengyou Liu, Xiaoxin Guo, Gang Du, La Yan, Zehong Zhang, Zhendong Zhong, Haiyang Chen

**Affiliations:** ^1^ Department of Neurology, National Clinical Research Center for Geriatrics, West China Hospital Sichuan University Chengdu Sichuan China; ^2^ Laboratory of Metabolism and Aging Research, National Clinical Research Center for Geriatrics, West China Hospital Sichuan University Chengdu Sichuan China; ^3^ MOE Key Laboratory of Gene Function and Regulation, Guangdong Province Key Laboratory of Pharmaceutical Functional Genes, State Key Laboratory of Biocontrol, School of Life Sciences Sun Yat‐sen University Guangzhou China

## Abstract

Sleep is essential for maintaining health. Indeed, sleep loss is closely associated with multiple health problems, including gastrointestinal disorders. However, it is not yet clear whether sleep loss affects the function of intestinal stem cells (ISCs). Mechanical sleep deprivation and *sss* mutant flies were used to generate the sleep loss model. qRT‐PCR was used to measure the relative mRNA expression. Gene knock‐in flies were used to observe protein localization and expression patterns. Immunofluorescence staining was used to determine the intestinal phenotype. The shift in gut microbiota was observed using 16S rRNA sequencing and analysis. Sleep loss caused by mechanical sleep deprivation and sss mutants disturbs ISC proliferation and intestinal epithelial repair through the brain–gut axis. In addition, disruption of SSS causes gut microbiota dysbiosis in *Drosophila*. As regards the mechanism, gut microbiota and the GABA signalling pathway both partially played a role in the *sss* regulation of ISC proliferation and gut function. The research shows that sleep loss disturbed ISC proliferation, gut microbiota, and gut function. Therefore, our results offer a stem cell perspective on brain–gut communication, with details on the effect of the environment on ISCs.

## Graphical abstract

Our findings show that sleep loss impairs intestinal stem cell function and gut homeostasis through the brain–gut axis in *Drosophila*. This study demonstrates that the disruption of SSS (a sleep‐promoting factor in *Drosophila*) impaired ISC functions partially through gut microbiota and the GABA signalling pathway.

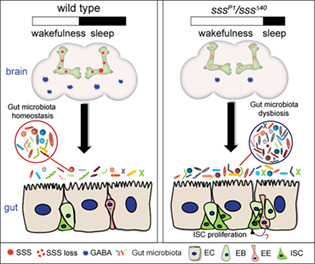

## 1 INTRODUCTION

Sleep is an essential requirement to maintain physiological functions and health.^1^, ^2^, ^3^ However, millions of people suffer from sleep deprivation worldwide,^4^, ^5^ which is now a recognized health concern in modern society.^6^ Sleep deprivation is associated with impaired memory and cognition^7^, ^8^ as well as severe health problems, including heart diseases, gastrointestinal disorders, diabetes, inflammatory responses, and mood disorders.^9^, ^10^, ^11^, ^12^, ^13^ Sleep deprivation also causes premature death in rats and flies.^3^, ^14^ Studies on sleep mechanisms and functions traditionally focused on the deficits of the nervous system,^15^, ^16^ but recent studies have demonstrated that the mechanism behind the lethal effect of sleep loss in mice and flies lies in the accumulation of reactive oxygen species (ROS) in the gut.^17^ Although inappropriate immune responses have been reported to play a role between sleep loss and gastrointestinal disorder,^18^ the exact mechanisms linking sleep deprivation with gut malfunction remain poorly understood. ROS balance regulates ISC proliferation,^19^, ^20^ and gut homeostasis depends on the accurate regulation of ISC activity.^21^, ^22^ Therefore, we aimed to investigate whether sleep deprivation affects ISC function and intestinal function.

To end this, we select the *Drosophila* model system. Rest behaviour in *Drosophila* is considered as a sleep state and a sleep episode is defined as periods of inactivity lasting at least 5 min.^23^, ^24^ And the core sleep regulatory mechanisms, including the neurotransmitter/neuropeptide system, ion channels, and the circadian clock network, are evolutionarily conserved from *Drosophila* to mammals.^3^, ^24^, ^25^ Various methods applied to *Drosophila* can be used to induce short‐term acute sleep deprivation and long‐term chronic sleep deprivation, including genetic manipulations, thermogenetic approaches, and mechanical stimulation.^23^, ^26^, ^27^, ^28^ Therefore, *Drosophila* is now widely used as a model to study sleep mechanisms and sleep function. In addition to the structural and functional similarity to mammalian small intestines, the *Drosophila* midgut is a powerful system for studying the functions of ISCs because of its simple genetic manipulation, well‐defined stem cell lineage, and easy‐to‐observe intestinal function. The *Drosophila* midgut is characterized by simple cellular components: ISCs, identified by the expression of the Notch ligand Delta (Dl) and the transcription factor Escargot (Esg); progenitor cells (enteroblasts (EBs) and enteroendocrine mother cells (EMCs)); polyploid absorptive enterocytes (ECs), expressing the transcription factor Pdm‐1; and diploid secretory enteroendocrine cells (EEs), expressing the transcription factor Prospero (Pros). Located in the basement membrane of the gut, *Drosophila* ISCs proliferate to self‐renew, and generate EBs or EMCs depending on the activity of the Notch signalling and differentiate into ECs or EEs.^22^, ^29^, ^30^ ISCs maintain intestinal homeostasis and regeneration through cell division and differentiation. The number of ISCs and progenitor cells in young and unchallenged intestines is relatively small and remains quiescent, and ISCs proliferate in response to tissue injury while avoiding over‐proliferation.^31^, ^32^ Dysregulation of ISCs is closely related to ageing, tumours, and intestinal disorders.^33^

Mutations of several sleep regulators in *Drosophila* induce decreased sleep, including mutations in *redeye* (*rye* ^*T227M*^), *insomniac* (*inc* ^*2*^), dopamine transporter (*DAT* ^*fmn*^), and *sleepless* (*sss* ^Δ40^).^17^ Among them, the *sleepless* (*sss*) mutant displayed the most significant sleep loss,^17^ becoming a suitable model to study the effect of sleep loss. The *sss* gene was identified as a sleep‐promoting factor in *Drosophila* through a forward genetic screen for sleep regulation.^28^ The *sss* gene encodes a small, glycosylphosphatidylinositol (GPI)–anchored membrane protein which regulates the Shaker‐dependent potassium current channel.^34^ The loss of SSS protein severely inhibits sleep (approximately an 80% reduction in sleep time), and sleep rebound is not obvious after sleep deprivation. Consistent with extreme sleep reduction, *sss* ^P1^ (a P‐element insertion in the *sss* gene)^28^ flies also exhibited a shorter lifespan. In addition, the activity records showed that *sss* ^P1^ flies have weak behavioural rhythmicity, while the circadian rhythm protein PERIOD fluctuates regularly and daily in clock cells.^28^ Therefore, the rhythmicity of *sss* mutants needs further study. Moreover, *sss* ^P1^ flies display uncoordinated behaviour such as the leg‐shaking phenotype. Thus, their climbing abilities are weakened to some extent, while the effect on flying and mating is very little.^35^ Interestingly, recent research found that *sss* mutants display ROS accumulation in the gut, causing premature death in *Drosophila*,^17^ suggesting that *sss* may play a role in the effects of sleep on the gut. Although the loss of SSS function has been reported as increasing the division rate of germ‐line stem cells in *Drosophila*,^36^ the function of the SSS protein in ISCs remains unknown.

This study found that sleep loss caused by *sss* mutation disturbed ISC proliferation, gut microbiota, and the normal digestive function of the intestine in *Drosophila*. Additionally, sleep loss regulated ISC proliferation partially through gut microbiota and the GABA signalling pathway. Therefore, this study revealed the effects of sleep on the gut from a stem cell perspective and improved our understanding of the regulation of stem cells by environmental signals.

## 2 MATERIALS AND METHODS

### 2.1 *Drosophila* lines and husbandry

The following fly lines were obtained from the Bloomington *Drosophila* stock centre (BDSC): *w* ^*1118*^ (BDSC# 3605), Canton‐S (CS) (BDSC# 64349), *UAS‐sss* (BDSC# 30866), *sss* RNAi (BDSC# 58061), *sss* ^P1^/*CyO* (BDSC# 16588), *sss* ^Δ40^/SM6a (BDSC# 30865), gabat^PL00338^ (BDSC# 19461), *elav‐Gal4* (BDSC# 8760) and *nSyb‐Gal4* (BDSC# 51635). The transgenic *Drosophila* line *sss‐GFP*/*CyO* was constructed in our laboratory.

The *esg‐GFP/CyO*, *tub‐Gal4*, *UAS‐lacZ*, and *actin* ^*ts*^ *‐Gal4* fly lines were kindly donated by Dr. Allan Spradling. The *Drosophila* lines used in this study are listed in Table S1.

Flies were kept at room temperature with 65% humidity and under 12:12 h light: dark cycles (12:12 LD) unless otherwise stated. The intestinal phenotypic observation and functional experiments were performed in the ZT0‐ZT6 period (Zeitgeber Time 0, when lights are turned on), and the experimental and control groups were kept in line. *Drosophila* stocks were maintained on a standard cornmeal‐agar medium (1 L food is composed of sucrose 80 g, cornmeal 50 g, glucose 20 g, yeast 18.75 g, agar 5 g, propionic acid 30 mL, dissolved in water). All experiments were performed using mated female flies (10–14 days old).

### 2.2 Generation of knock‐in fly lines

Two constructs were generated, one with two sgRNAs and the other with a homologous recombination sequence, to obtain the knock‐in line. The two distinct sgRNAs were used to generate the deletion in the genome regions of interest. The sgRNA sequence was synthesized in vitro and sub‐cloned into a PMD18T vector to obtain the U6 promoter. The U6 promoter and sgRNA were amplified from the PMD18T vector by PCR. Two PCR products with the U6 promoter and sgRNA were sub‐cloned together into the PCR8 vector using the Golden Gate assembly and then recombined into the attB vector using the LR recombination reaction to generate the sgRNA construct. The 5′ homologous arm (~1 KB), the eGFP, and the 3′ homologous arm (~1 KB) were inserted into the PASK vector to generate the homologous recombination construct. The 5′ homologous arms and 3′ homologous arms were used for homologous recombination repair and the eGFP was introduced before stop codn of *sss* gene by homologous recombination. The 3 × P3‐RFP was also introduced for screening. The used sgRNAs were designed by http://targetfinder.flycrispr.neuro.brown.edu/ and they are the following:

Target1‐*sss*‐sgRNA: GCTCTCTCTTTCAGCGTACGAGG.
Target2‐*sss*‐sgRNA: CTCTCTTTCAGCGTACGAGGTGG.

### 2.3 Sleep monitoring and sleep assays

Individual flies were placed into glass tubes containing sucrose‐agar food and entrained at 25°C in 12:12 LD. Sleep was tracked by collecting motor activity data using *Drosophila* activity monitoring systems (DAM2, TriKinetics). When the fly moves back and forth, it disturbs the infrared beams of the machine, leaving a record. And when the fly was inactive for 5 min, it was recorded as a sleep episode.^24^ The sleep data were collected and converted using the software DAM File Scan, and then analysed using Microsoft Excel in combination with Prism 8.0 (GraphPad).

Sleep time was presented as a percentage of total time. Student's *t* test for statistical difference in total sleep, daytime sleep, night‐time sleep, and rebound sleep among genotypes.

### 2.4 Sleep deprivation

Mechanical sleep deprivation was performed at 25°C using a Multipurpose Shaker QB‐206. After eclosion, flies were raised at room temperature (12:12 LD) for 10 days, then flies were placed into shaking tubes containing standard food. The intensity of the shaker was set to 12. The guts were dissected after 24 h stimulation.

Rebound sleep (Δsleep) was recorded after one night of mechanical sleep deprivation. It was determined for each fly by subtracting the sleep on the morning before deprivation (ZT0‐ZT4) from the sleep on the morning (ZT0‐ZT4) after deprivation.^17^, ^37^

### 2.5 Immunofluorescence and microscopy

*Drosophila* midguts and brains were dissected in PBS and then fixed with 4% paraformaldehyde for 30 min. Tissue fixation was performed in the dark and washed 3 times (10 min each) with PBS containing 0.1% Triton X‐100 (PBST). Next, the midguts and brains were blocked using 0.5% BSA for 30 min at room temperature, and then, they were incubated overnight at 4°C with primary antibodies diluted in PBST. Next, the midguts and brains were washed 3 times with 0.1% PBST as mentioned above and incubated with a mixture of secondary antibodies and DAPI for 2 h at room temperature. The same washing was performed as the final step. All the primary antibodies are listed in Table S2.

Leica TCS‐SP8 confocal microscope was used to acquire all the immunofluorescence images. The Leica Application Suite X (LAS X), Adobe Photoshop CC 2021 and Adobe Illustrator 2020 were used to assemble the images.

### 2.6 Dihydroethidium (DHE) staining

DHE staining was performed to observe the levels of ROS in tissues.^19^ Guts were dissected in PBS and incubated in Schneider's medium mixed with 30 μM DHE (MKbio, #MX4812) and Hochest 33342 (10 μg/mL) for 10 min. The guts were then washed three times in Schneider's medium at room temperature, and images were immediately captured by a microscope.

### 2.7 Bromophenol blue treatment

Bromophenol blue assay was performed to observe the acid–base homeostasis in the guts as previously described.^38^, ^39^ The 200 μL of 2% Bromophenol blue sodium (pH indicator, Sigma, B5525) was added to the vial containing the normal food, and several holes were made on the surface of the food using a pipet tip to ensure full absorption. After 24‐h feeding, images were taken soon after gut dissection.

### 2.8 Fly excretion measurement

The fly excretion measurement was performed after starving the flies for 2 h, and then they were placed into 2% bromophenol blue food vials whose walls were surrounded by chromatography paper. The deposits on the paper were imaged and quantified after 24 h. Each group includes 15 flies.

### 2.9 Food intake assay

Colorimetric estimation of food consumption was performed as previously described.^40^ Flies were transferred onto food containing 2.5% (w/v) FD&C Blue #1, 5% sucrose, and 2% agar, and fed ad libitum. The flies were washed with PBS after 24‐h feeding, the body of each fly was separated from its head, and the bodies of 10 flies were homogenized in 200 mL cooled 0.1% PBST and centrifuged at 10,000 rpm for 10 min. The 50 mL supernatant was collected and the absorbance was measured at 625 nm (A625) using a microplate reader. Flies fed on standard cornmeal‐agar food were used as controls.

### 2.10 ‘Smurf’ assay

FD&C blue #1 dye was added to the standard cornmeal‐agar food at a concentration of 2.5% (w/v). Flies were subjected to 2‐h starvation, then fed on the dyed food for 12 h, and observed. Smurf flies with the blue colour visible outside the digestive tract were counted.^41^

### 2.11 Bleomycin treatment

Chromatography papers were cut into 3.5 × 5.5 cm strips and saturated with 25 μg/mL bleomycin (Aladdin, B107423) dissolved in 5% (w/v) sucrose. Flies were subjected to 2‐h starvation, and every 20 flies were transferred into a vial containing a chromatography paper saturated with bleomycin solution or 5% (w/v) sucrose solution serving as control. After 24‐h bleomycin feeding, flies were transferred to tubes containing standard food for recovery. Tissues were dissected after recovery for 1 or 3 days.

### 2.12 RT‐qPCR

Guts and heads were dissected in pre‐cold diethyl pyrocarbonate (DEPC)‐treated water‐PBS. Total RNA was extracted from the dissected tissues using the RNA‐easy Isolation Reagent (Vazyme), and 1 μg template RNA was used to generate cDNA by reverse transcription using the *Evo* M‐MLV RT Kit (Accurate Biology). The cDNA was used to perform a quantitative polymerase chain reaction (qPCR) using SYBRGreen (Vazyme) by a CFX96™ Real‐time PCR System (BIO‐RAD). The relative expression of genes was calculated by the 2^−ΔΔCt^ method and normalized to that of the housekeeping gene Rp49. The following primers were used:

SSS L: 5′ TGCATGATGGAAAGTTCAGG 3′;
SSS R: 5′ AGCCAAGATACTGCCACTGC 3′;
Rp49 L: 5′ ACTTCATCCGCCACCAGTC 3′;
Rp49 R: 5′ ATCT CGCCGCAGTAAACG 3′.

### 2.13 Isoguvacine (IG) feeding

The GABA_A_ receptor agonist IG (MCE) was dissolved in DMSO and added to the regular food medium at a final concentration of 1 mM. Flies within 3 days after eclosion were collected and placed into vials containing food mixed with IG and transferred to fresh vials every 2 days until 10 days to activate the GABAergic signalling. The control food was mixed with the same volume of DMSO.

### 2.14 Bacterial culture and colony count

Each fly was disinfected in 95% ethanol for 1 min, then the gut was dissected, homogenized in 200 μL 1xPBS, and the volume was raised to 1 mL. The sample was centrifuged for 30 s at 1000 rpm and the supernatant was collected. One hundred microlitres supernatant was plated on nutrient‐agar (NA) plates. The bacteria were incubated on NA plates at 30°C for 36–48 h followed by a colony count.^42^, ^43^

### 2.15 Antibiotic treatment

Antibiotic media was prepared by utilizing a previously published antibiotic cocktail^42^, ^44^: cornmeal‐agar food with a final concentration of 100 μg/mL Ampicillin, 50 μg/mL Kanamycin, 50 μg/mL Tetracyclin, and 200 μg/mL Rifampicin. Flies were treated with antibiotic media for 5 days to eliminate pathogenic‐like bacteria overgrowth.

### 2.16 16S rRNA sequencing and analysis

The bacterial DNA was obtained from 15 dissected guts from female (10–14 days old) wild‐type and *sss* ^*P1*^ */sss* ^*Δ40*^ flies using MagPure Soil DNA LQ Kit (Magen). Four biological replicates per group were used. The PCR amplification of the V3‐V4 hypervariable regions of the bacterial 16S rRNA gene was performed in a 25 μL reaction solution using universal primer pairs (343F: 5′ TACGGRAGGCAGCAG 3′; 798R: 5′ AGGGTATCTAATCCT 3′). The reverse primer contained a sample barcode and both primers were related to an Illumina sequencing adapter. The PCR products were purified using Agencourt AMPure XP beads (Beckman Coulter Co., USA) and quantified using a Qubit dsDNA assay kit. Sequencing of the 16S amplicon was performed by OE Biotech Co., Ltd. on an Illumina MiSeq. Using the Trimmomatic program,^45^ paired‐end reads were preprocessed to find and remove ambiguous bases (N). Using the sliding window trimming technique, it was also utilized to remove low‐quality sequences with an average quality score below 20. After trimming, paired‐end reads were assembled with FLASH software.^46^ Reads with 75% of bases above Q20 were kept using QIIME software (version 1.8.0). Using the VSEARCH software, clean reads were subjected to primer sequence removal and clustering to produce operational taxonomic units (OTUs) with a 97% similarity cut‐off. The representative read of each OTU was chosen using the QIIME package. All representative reads were annotated and blasted against the Silva database (Version 132) using the RDP classifier (confidence threshold of 70%).^47^

The microbial diversity in the gut content was estimated using the alpha diversity that includes the Shannon index and Simpson index,^48^ while beta diversity was estimated using the principal component analysis (PCA). Microbial multivariate statistical analysis was performed to calculate the differential bacteria (including OTUs, phylum, class, order, family, genus, and species) between different subgroups by the statistical algorithm one‐way ANOVA and to perform the differential species heat map.

### 2.17 Statistical analysis

Statistical analysis was performed using Prism 8 (GraphPad Software). Differences between groups were assessed using unpaired two‐tailed Student's *t* tests and Fisher's exact test. In brief, Mean and SEM is used in the interaction graphs and sleep data, and in the other dot plots, we used Mean and SD. A value of *p* < 0.05 was considered statistically significant.

### 2.18 Data and software availability

Prism 8.0 (GraphPad) was used in this study and is available at https://www.graphpad.com/. The Adobe Photoshop CC 2021 and Adobe Illustrator 2020 are available at https://www.adobe.com/products/catalog.html. The Leica Application Suite X (LAS X) is available at https://www.leicamicrosystems.com/products/microscope-software/p/leica-las-x-ls/. All 16S rRNA datasets are publicly available in Sequence Read Archive (SRA) BioProject: PRJNA874194.

## 3 RESULTS

### 3.1 Sleep deprivation disturbs ISC proliferation and intestinal epithelial repair

**FIGURE 1:**
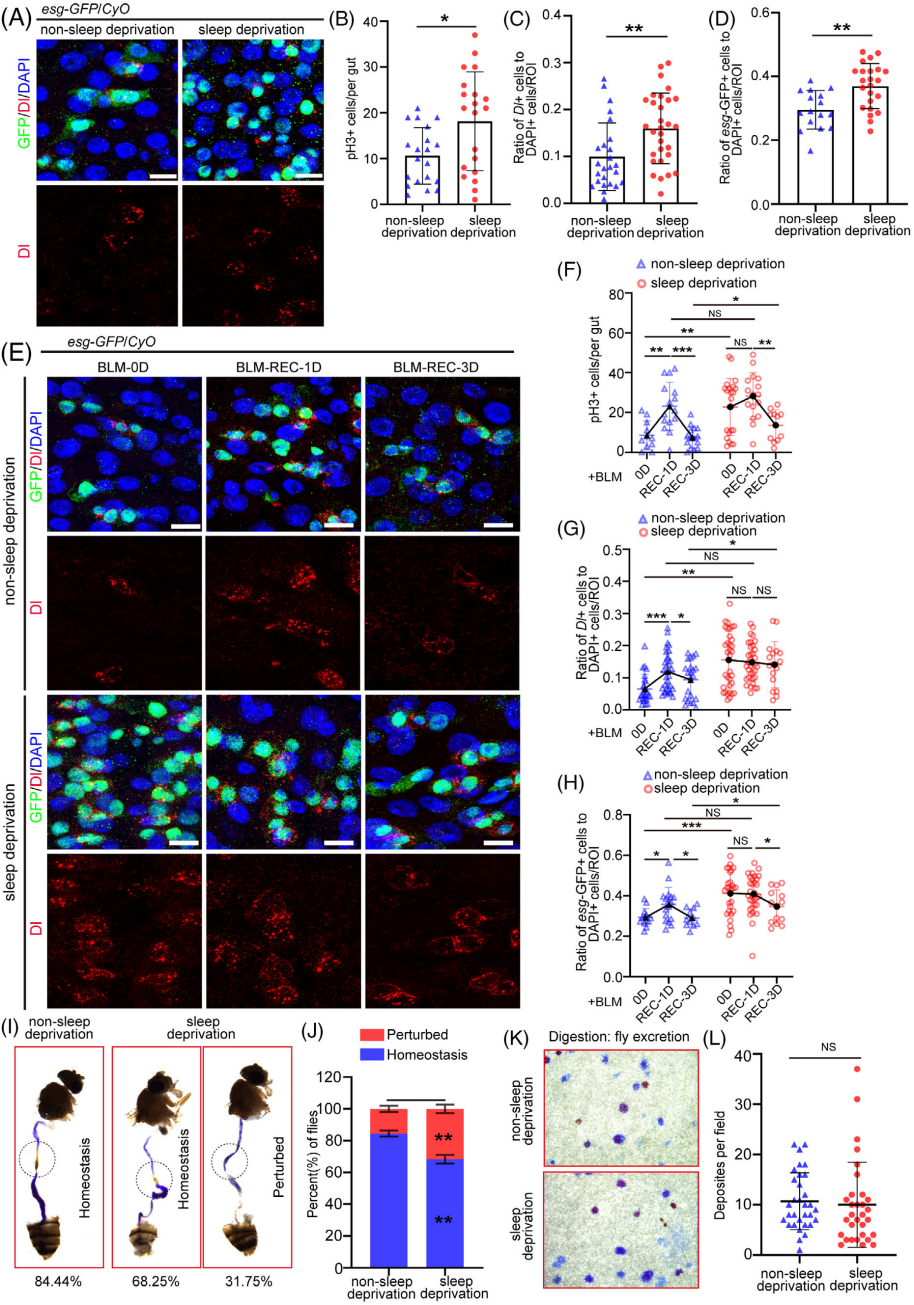
Sleep deprivation disturbs ISC proliferation and intestinal epithelial repair. (A) Representative immunofluorescence images of sleep deprivation and non‐sleep deprivation flies from the R4 or R5 region of the midguts with GFP and Dl staining. *esg*‐GFP (green) was used to visualize ISCs and EBs, and Dl staining (red) identifies ISCs. (B) Quantification of pH3^+^ cell number in the gut of sleep deprivation and non‐sleep deprivation flies. Each dot corresponds to one gut. Mean and SD. (C and D) Quantification of the ratio of Dl^+^ cells (C) and *esg*‐GFP^+^ cells (D) to DAPI^+^ cells in sleep deprivation and non‐sleep deprivation flies. Each dot represents one ROI in the R4 or R5 region of the midgut. ROI =3.4 × 10^4^ μm^2^ area. Mean and SD. (E) Representative immunofluorescence images in the guts of BLM‐0D, BLM‐REC‐1D, and BLM‐REC‐3D flies with sleep deprivation and non‐sleep deprivation. (F) Quantification of the number of pH3^+^ cells per gut. Each dot corresponds to one gut. Mean and SD. (G and H) Quantification of the ratio of Dl^+^ cells (G) and *esg*‐GFP^+^ cells (H) per ROI. Each dot corresponds to one ROI. Mean and SD. (I and J) Representative images (I) and quantification (J) of the percentage of gut acid–base homeostasis of sleep deprivation flies and non‐sleep deprivation flies. The CCR region is indicated by a circle. The type of the GI tract of flies fed with Bromophenol blue include ‘Homeostasis’ (CCR area: yellow) and ‘Perturbed’ (CCR area: blue). Error bars represent the SD of three independent experiments. *N* = 15 flies per group. (K and L) Representative images (K) and quantification (L) of fly excretion deposits from sleep deprivation flies and non‐sleep deprivation flies fed with Bromophenol Blue. Each sample contains three independent experiments. Excretions are quantified for 12 fields in each group of 15 flies. Mean and SD. Scale bar = 10 μm. DAPI‐stained nuclei are shown in blue. Student's *t* test, *p < 0.05, **p < 0.01, ***p < 0.001, NS = not significant.

To investigate the effects of sleep loss on the regulation of ISC functions and gut epithelial homeostasis, *Drosophila* was subjected to 24‐h acute sleep deprivation by mechanical stimuli (Figure S1A,B) as previously described.^24^ Phosphorylated Histone 3‐positive (pH3^+^, a mark of mitosis) cells, *esg*‐GFP‐positive (*esg*‐GFP^+^) cells, and Delta‐positive (Dl^+^, indicating active ISCs) cells were analysed to evaluate ISC proliferation. Results showed that sleep deprivation led to an increase in the ISC proliferation rate of *Drosophila* (Figures 1A–D and S1C). Since ISCs are responsible for the repair of the injured epithelium by differentiating into mature intestinal cells,^32^ the effect of sleep deprivation on ISC‐mediated intestinal epithelial repair was further investigated. An ‘injury‐and‐recovery’ model^49^ was used by feeding the flies with bleomycin (BLM) (Figure S1D). The non‐sleep deprivation flies showed a pattern of an initial increase and then decrease in ISCs during midgut regeneration, while the activated ISCs in sleep‐deprived flies did not increase in response to the intestinal injury in BLM‐REC‐1D and they did not timely return to a quiescent state in BLM‐REC‐3D (Figures 1E–H and S1E–H). Therefore, sleep deprivation delayed ISC‐mediated epithelium repair in *Drosophila*. Next, the gut acid–base homeostasis and excretion^50^ of *Drosophila* were observed to evaluate whether intestinal function was affected by sleep loss. The acid–base homeostasis was disrupted in sleep‐deprivation flies (Figure 1I,J), while fly excretion was not impaired (Figure 1K,L). In summary, the above results suggested that sleep deprivation impaired intestinal homeostasis in *Drosophila*.

### 3.2 Sleep‐promoting factor SSS regulates ISC proliferation and intestinal epithelial repair in *Drosophila*

**FIGURE 2:**
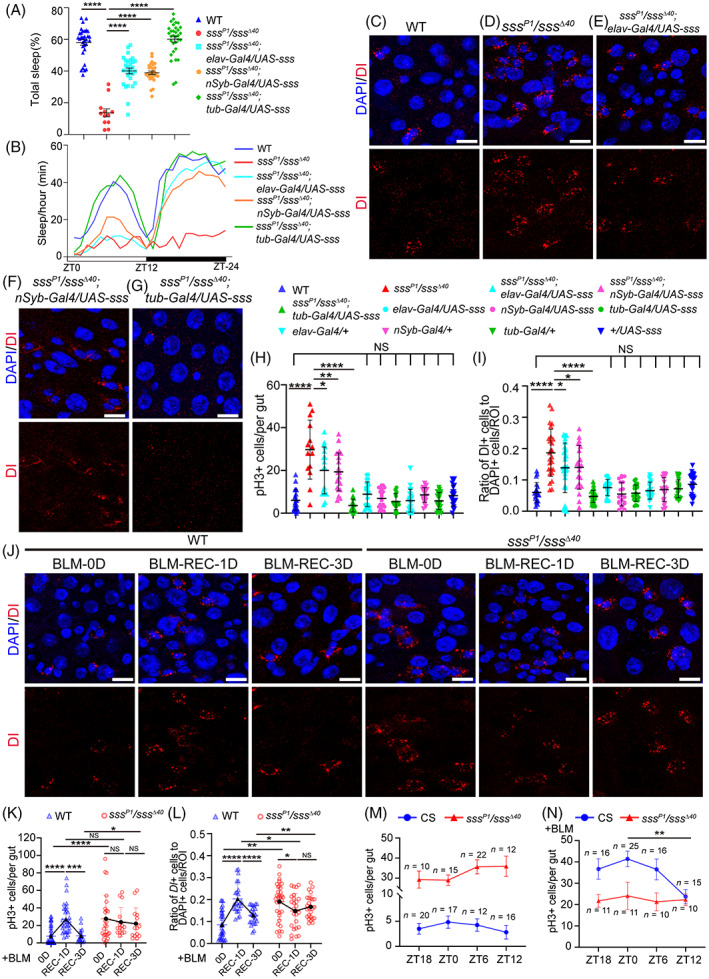
SSS regulates ISC proliferation and intestinal epithelial repair in *Drosophila*. (A and B) Total sleep (A) and sleep per hour (B) in WT, the *sss* ^P1^/*sss* ^Δ40^ and rescued flies. Mean and SEM. (C–G) Re‐expression of *sss* (*UAS*‐*sss*) in the neurons (*elav*‐*Gal4*), neurons (*nSyb*‐*Gal4*) and whole body (*tub*‐*Gal4*) in *sss*‐deficient flies rescued the ISC proliferation defect. Representative Dl (red; ISC marker) immunofluorescence images from the midgut R4 or R5 sections with the indicated genotypes (C)–(G). (H) Quantification of the number of pH3^+^ cells in the gut of flies with the indicated genotypes (C)–(G). Each dot corresponds to one gut. Mean and SD. (I) Quantification of Dl^+^ cell ratio per 3.4 × 10^4^ μM^2^ in the midgut of flies with the indicated genotypes. Each dot corresponds to one ROI. Mean and SD. (J) Representative immunofluorescence images of the R4 or R5 region of the midgut of the WT and the *sss* ^P1^/*sss* ^Δ40^ flies in BLM‐0D, BLM‐REC‐1D, and BLM‐REC‐3D. Dl staining (red) identifies ISCs. (K) Quantification of pH3^+^ cell number of flies with the indicated genotypes. Each dot represents one gut. Mean and SD. (L) Quantification of the ratio of Dl^+^ cells per ROI in the midgut of the experiment (J). Each dot represents one ROI. Mean and SD. (M and N) ISC division rhythm in the 12:12 h LD cycle. ZT is defined by the light signal, with ZT0 pointing to lights on and ZT12 pointing to lights off. Line graphs show the trend in pH3^+^ cell change in CS flies (blue curve) and *sss* ^P1^/*sss* ^Δ40^ flies (red curve) with (N) and without (M) BLM treatment. Significant differences are detected between time points ZT0 and ZT12 in CS flies (N). Mean and SEM. Scale bar = 10 μm. DAPI‐stained nuclei are shown in blue. WT = wild type. Student's *t* test, *p < 0.05, **p < 0.01, ***p < 0.001, ****p < 0.0001, NS = not significant.

Genetic mutants with reduced sleep in *Drosophila* were next explored because chronic deprivation is difficult to achieve with mechanical stimuli. The mutant with the loss of SLEEPLESS (SSS), the sleep‐promoting factor, showed a significant (>80%) decrease in sleep.^28^ Thus, it is a suitable model for investigating the potential effects of sleep loss. Flies with decreased level of SSS was obtained using a combination of two *sss* alleles (*sss* ^P1^/*sss* ^Δ40^). This transheterozygote mutant showed a strong sleep deprivation effect (Figure 2A,B). Consistent with the phenotype we observed in wild‐type flies treated with mechanical stimuli, the number of pH3^+^ cells and Dl^+^ cells in *sss* ^P1^/*sss* ^Δ40^ transheterozygote flies were increased compared with the control flies (Figures 2C,D,H,I and S2B). Moreover, the increased ISC proliferation rate in *sss* mutant flies was significantly rescued by the re‐expression of SSS cDNA driven by *tub*‐*Gal4*, *elav*‐*Gal4*,^51^ and neuron‐specific *nSyb‐Gal4* ^52^ (Figures 2E,I and S2A,B). These results demonstrated that the increased ISC proliferation in the *sss* ^P1^/*sss* ^Δ40^ flies was indeed caused by the loss of SSS protein and that *sss* regulated ISC proliferation in *Drosophila*. To exclude the effect of SSS protein deletion on the developmental stage, we used conditional temperature‐sensitive driver *actin* ^*ts*^ to drive *sss* RNAi. The sleep data indicated that the knockdown group had less sleep than the control group, but we also discovered that it had some influence on daytime sleepiness (Figure S2C), perhaps as a result of the delay between gene transcription and translation. The pH3 staining of *Drosophila* carrying *actin* ^*ts*^‐ driven *sss* RNAi showed an increase in ISC proliferation compared with the control flies (Figure S2D), which was consistent with the phenotype of the *sss* mutants. And during midgut regeneration, the wild‐type flies showed the dynamics of the quiescent and active state of ISCs (Figures 2J,K and S2E), and the ISCs in midguts of *sss* ^P1^/*sss* ^Δ40^ *Drosophila* were always in an abnormally activated state (Figures 2J–L and S2E), which was detrimental to intestinal repair. Thus, sleep loss caused by SSS deficiency repressed ISC‐mediated epithelium repair in *Drosophila*. Previous studies showed that the division of ISCs is rhythmically influenced by local, environmental, and systemic factors.^53^, ^54^ In addition, the daily rhythms are essential for gut homeostasis.^55^ Sleep–wake cycles are important to ensure the normal function of the organs. Therefore, the effect of sleep loss on the division rhythm of ISCs was explored. The ISC division in CS flies under the 12:12 h dark: light cycle showed a rhythm trend (Figure 2M), which was significant under BLM condition (Figure 2N). The *sss* mutants showed a loss of rhythmic variation under both normal food and BLM conditions (Figures 2M,N and S2F). These results suggested that SSS also played a regulatory role in the division rhythm of ISCs in *Drosophila*.

### 3.3 Depletion of SSS leads to the functional decline of *Drosophila* gut

**FIGURE 3:**
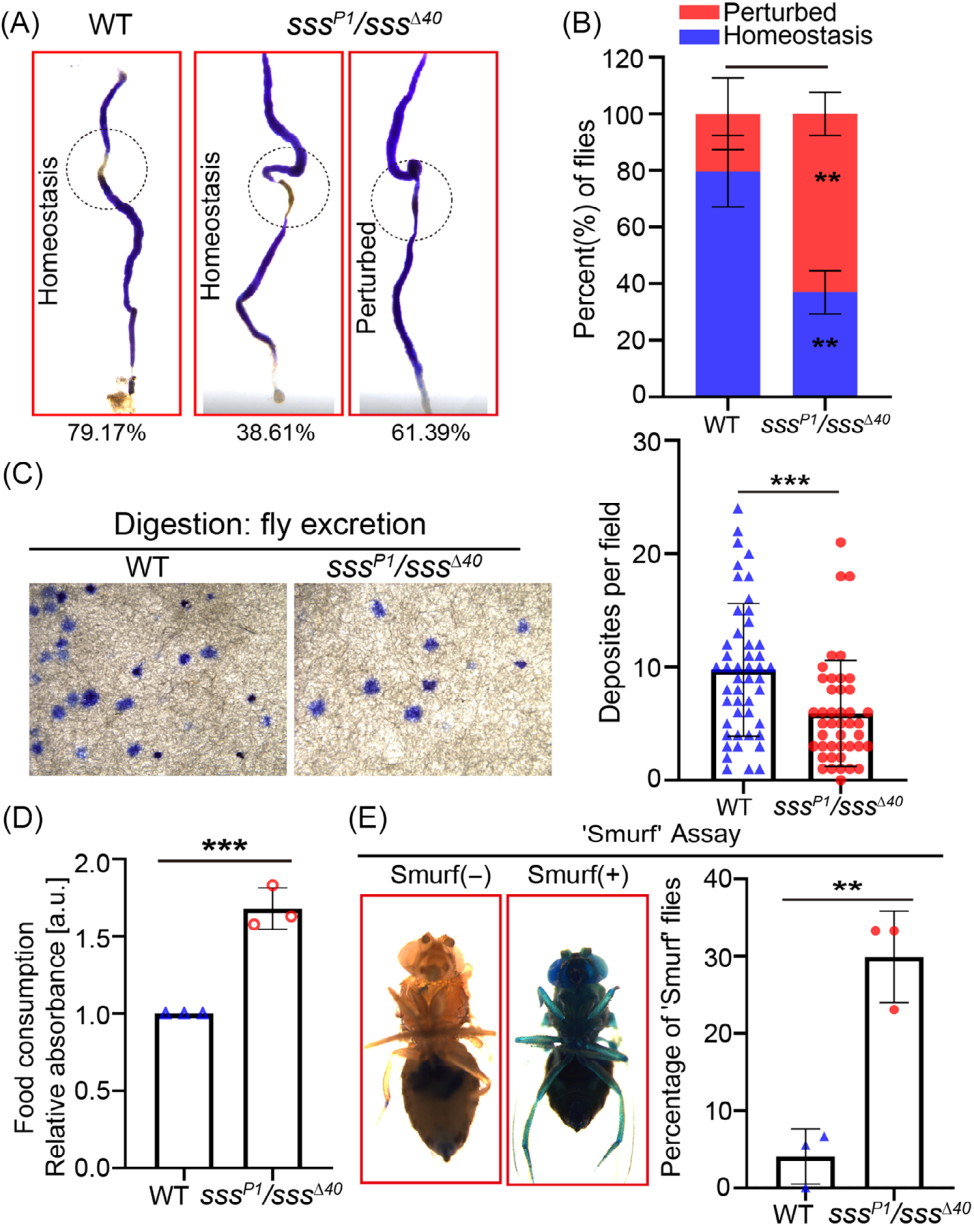
Depletion of SSS leads to the functional decline of the *Drosophila* gut. (A and B) Representative images (A) and quantification (B) of the percentage of intestinal acid–base homeostasis of the WT and *sss* ^P1^/*sss* ^Δ40^ flies. The circle shows the CCR area. Error bars represent the SD of three independent experiments. (C) Representative images (left) and quantification (right) of fly excretions from the WT and *sss* ^P1^/*sss* ^Δ40^ flies fed with Bromophenol Blue. Mean and SD. (D) Measurements of food consumption of the WT and *sss* ^P1^/*sss* ^Δ40^ flies through a colorimetric estimation after the treatment with non‐absorbed blue dye. Error bars represent the SD of three independent experiments. (E) Representative images (left) and quantification (right) of the percentage of ‘Smurf’ flies in WT and *sss* ^P1^/*sss* ^Δ40^ flies. *Drosophila* with and without blue dye visible outside the digestive tract were recorded as Smurf (+) and Smurf (−) flies, respectively. Error bars represent the SD of three independent experiments. WT = wild type. Student's *t* test, **p < 0.01, ***p < 0.001.

The appropriate proliferation and differentiation of ISCs are important in the maintenance of gut function and intestinal epithelial repair.^31^ Since sleep loss induced by SSS depletion leads to abnormal ISC proliferation, the effect of sleep loss on intestinal function in *Drosophila* was evaluated. The gut in healthy *Drosophila* showed intestinal compartmentalization and acid–base homeostasis to maintain normal digestive function.^38^ However, flies with *sss* depletion showed a remarkable decline in intestinal digestive functions, including the loss of acid–base homeostasis (Figure 3A,B), a decline in food excretion (Figure 3C) and an increase in food intake (Figure 3D). The evaluation of the intestinal barrier function also showed a significant increase in intestinal permeability in that of the *sss* mutants (Figure 3E). Taken together, these results revealed that flies with sleep loss showed a remarkable decline in intestinal function. The intestinal outcomes of these two sleep deprivation methods were consistent, so we next explored whether the effects of mechanical sleep deprivation and SSS loss are linked. We found that overexpression of SSS using *elav‐Gal4* could not rescue the defect of ISC over‐proliferation caused by mechanical sleep deprivation (Figure S2G). And the qPCR analysis showed no significant change in *sss* expression with mechanical sleep deprivation (Figure S2H). This result indicated that mechanical sleep deprivation induces ISC over‐proliferation in *Drosophila* midguts through an SSS‐independent mechanism.

### 3.4 SSS regulates ISC proliferation through the modulation of the brain–gut axis

**FIGURE 4:**
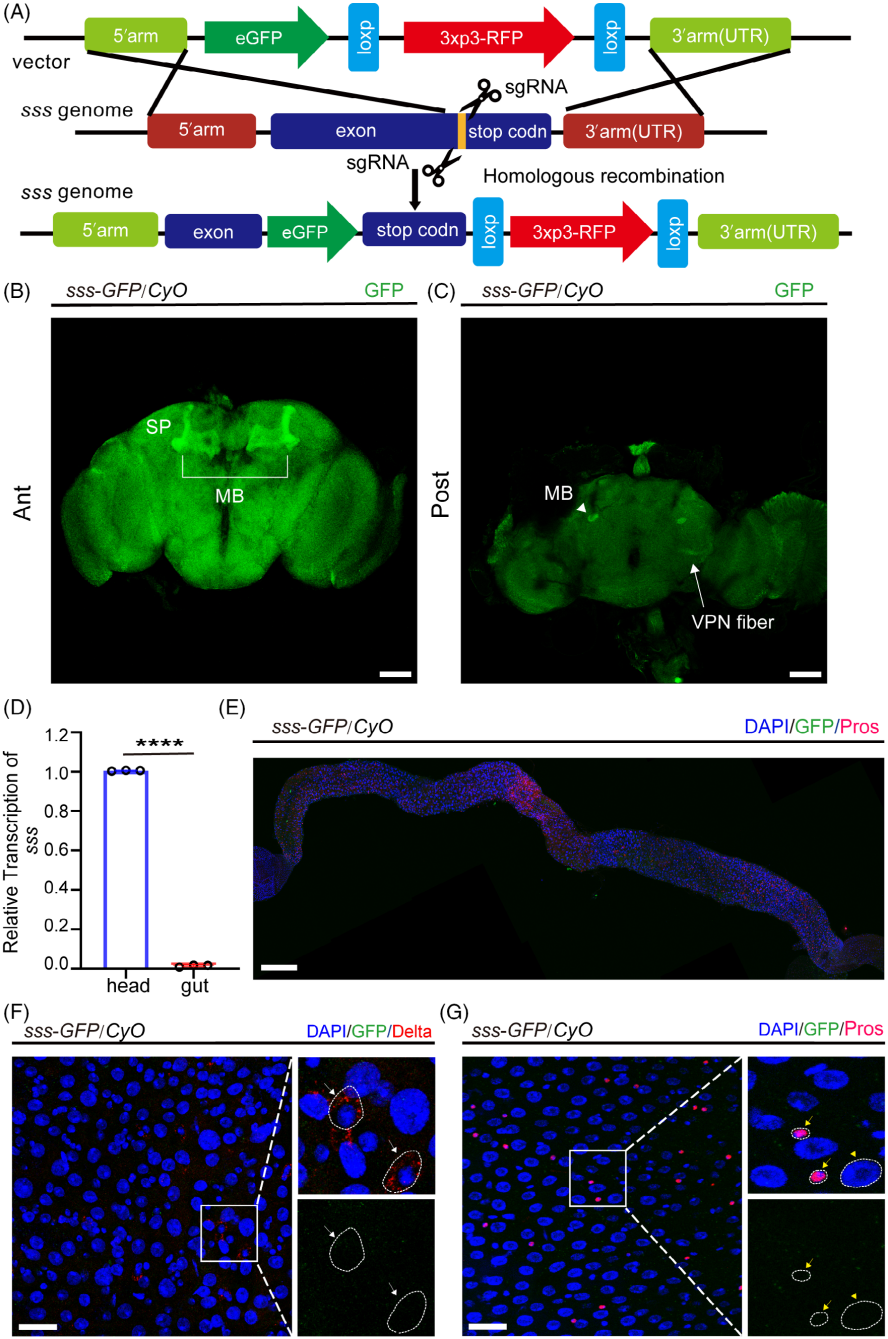
Expression pattern of the endogenous SSS protein in *Drosophila*. (A) Strategy for the construction of *Drosophila* endogenous *sss‐GFP* knock‐in line with the CRISPR/Cas9 system. (B and C) Immunofluorescence images of *sss‐GFP* (green) expression pattern in fly brain. SSS is expressed in MB (bracket and white arrowhead), SP, and VPN (white arrow). (D) *sss* relative mRNA expression between the heads and guts of wild‐type flies. Error bars represent the SD of three independent experiments. (E) Immunofluorescence images of the expression pattern of SSS (green) in the whole gut. Prospero staining (magenta) identifies EEs. Scale bar = 200 μm. (F and G) Representative images showing the expression of *sss‐GFP* (green) in various types of cells in the *Drosophila* intestine. Scale bar = 25 μm. The right image is a partial enlargement of the left panel. The upper part shows the Dl (red) staining, and the lower shows the GFP (green) staining. White arrows indicate ISCs (Dl^+^), yellow arrows indicate EEs (Pros^+^), and yellow arrowheads indicate ECs (large and polyploid). Scale bar = 10 μm. DAPI‐stained nuclei are shown in blue. Student's *t* test, ****p < 0.0001.

The endogenous SSS reporter strain, *sss‐GFP*, was generated using the CRISPR‐Cas9 knock‐in system to observe the expression pattern of endogenous SSS protein in *Drosophila* and investigate how *sss* plays a role in regulating ISC proliferation (Figure 4A). The immunofluorescence results showed that SSS was highly expressed in the mushroom bodies (MB), superior protocerebrum (SP), and visual projection neurons (VPN) fibres in the *Drosophila* brain (Figure 4B,C). This result is consistent with previous studies.^34^ In addition, no exact expression of SSS protein was found in the *Drosophila* gut, including ISCs (Dl^+^ cells), EEs (Pros^+^ cells), or ECs (polyploid cells) (Figure 4E,G). Moreover, the qPCR analysis showed that the mRNA transcription of *sss* was indeed highly expressed in the *Drosophila* brain but almost not expressed in the gut (Figure 4D). Moreover, we used a neuron‐specific Gal4, *nSyb‐Gal4*,^52^, ^56^, ^57^ to deplete SSS in neurons. The results showed that *nSyb‐Gal4*‐driven sss RNAi led to an increase in ISC proliferation compared to control flies (Figure S2I). The above results indicated that *sss* regulated ISC proliferation through a mechanism mediated by brain‐to‐gut communication. Therefore, combining the above factors, the SSS gut phenotype is the most likely outcome of the brain. Additionally, we found that *sss* mutants showed ROS accumulation in the gut, consistent with a previous report,^17^ but this phenomenon was only present in the anterior midgut and not the posterior midgut (Figure S2J). Increased regional ROS does not provide a satisfactory explanation for the phenotype of increased ISC throughout the gut. Thus, it implies that there are more mechanisms.

### 3.5 Disruption of SSS causes gut microbiota dysbiosis in *Drosophila*

**FIGURE 5:**
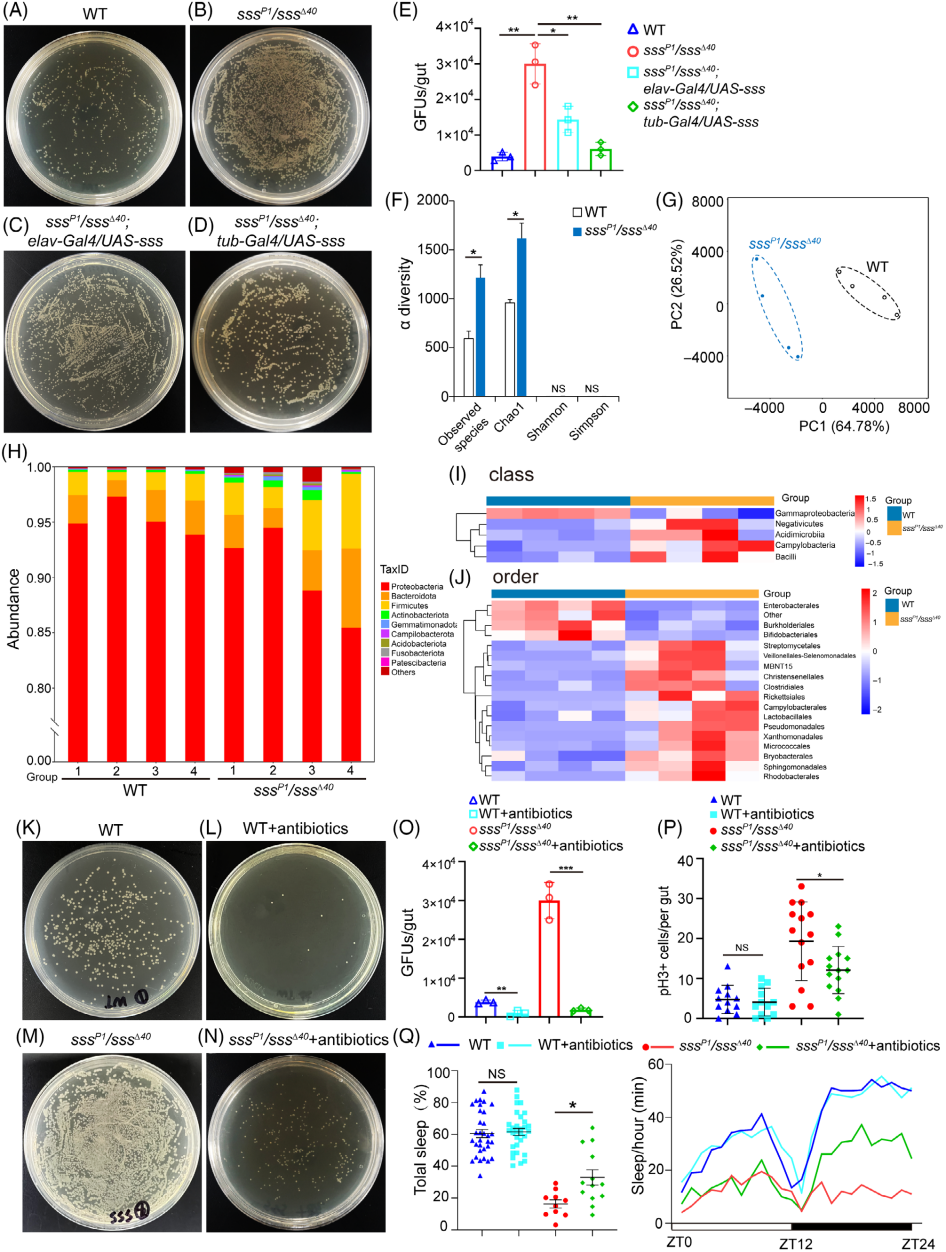
Disruption of SSS causes gut microbiota dysbiosis in *Drosophila*. (A–E) Representative images (A–D) and quantification (E) of the bacterial load in WT, *sss* ^P1^/*sss* ^Δ40^ flies, and rescued flies. The bacterial load in flies was determined by colony count. Intestine samples were cultured on NA plates at 30°C. Mean and SD. (F) α‐diversity of the bacterial species in gut samples from WT and *sss* ^P1^/*sss* ^Δ40^ flies by Wilcoxon rank‐sum test. *p < 0.05, NS = not significant. (G) PCA showing significant differences in the gut microbiota between WT and *sss* ^P1^/*sss* ^Δ40^ flies. (H) Relative abundances of bacterial phylum structure in gut samples of WT and *sss* ^P1^/*sss* ^Δ40^ flies. (I and J) Heatmap of the normalized relative abundance of OTUs significantly changed between WT and *sss* ^P1^/*sss* ^Δ40^ flies at the class level (I) and order level (J) by one‐way ANOVA, *p* < 0.05. (K–O) Representative images (K–N) and quantification (O) of the bacterial load in WT and *sss* ^P1^/*sss* ^Δ40^ flies fed with and without antibiotics. Mean and SD. (P) Quantification of pH3^+^ cell number in the gut of (K)–(N). Mean and SD. (Q) Total sleep (left) and sleep per hour (right) in WT and *sss* ^P1^/*sss* ^Δ40^ flies fed with and without antibiotics. Mean and SEM. WT = wild type, Student's *t* test, *p < 0.05 **p < 0.01, ***p < 0.001, NS = not significant.

Increasing evidence shows that the gut microbiota is closely related to various features of *Drosophila* physiology and intestinal homeostasis, including ageing, inflammatory responses, metabolic function, and social behaviour.^42^, ^44^, ^58^ Therefore, the effect of sleep loss on the gut microbiota in *Drosophila* was investigated. The simple bacterial culture proved that the number of colony‐forming units (CFUs) in the *sss* mutant significantly increased (Figure 5A,B,E). And the changes in the abundance of gut microbiota that were rescued in mutant flies by overexpressing of SSS (Figure 5C,E). This result indicated that sleep loss may cause microbiota dysbiosis. This hypothesis was further confirmed by performing 16S ribosomal RNA gene sequencing of the gut DNA isolated from *sss* ^P1^/*sss* ^Δ40^ and wild‐type flies. The gut microbiota of *sss* ^P1^/*sss* ^Δ40^ flies had a higher number of observed species and chao1 indexes compared to those of the control, while the α diversity of intestinal flora had no obvious difference (Figure 5F). In addition, PCA revealed a significant difference in microbiota composition between wild‐type and *sss* ^P1^/*sss* ^Δ40^ flies (Figure 5G). Then the phylum abundance analysis indicated that five previously described major gut bacteria (*Firmicutes*, *Bacteroidota*, *Proteobacteria*, *Acidobacteriota*, and *Actinobacteriota*)^59^ were found both in the wild‐type and *sss* ^P1^/*sss* ^Δ40^ flies (Figure 5H). The gut microbiota of the *sss* ^P1^/*sss* ^Δ40^ flies had increased levels of *Acidobacteriota* and *Campilobacterota* (Figure S3A). At the class level, *Campylobacteria*, *Bacilli*, *Negativicutes*, and *Acidimicrobiia* were more abundant, while *Gammaproteobacteria* was less abundant in *sss* ^P1^/*sss* ^Δ40^ flies (Figure 5I). Consistently, gram‐negative bacteria such as *Xanthomonadales*, *Sphingomonadales*, *Campylobacterales*, and *Pseudomonadales* were increased in *sss* ^P1^/*sss* ^Δ40^ flies. Specifically, *Bifidobacteriales*, an intestinal probiotic, were reduced in *sss* ^P1^/*sss* ^Δ40^ flies (Figures 5J and S3B–D, and Table S3). According to the 16S rRNA gene sequencing, sleep loss caused by *sss* mutants modulated the microbiome composition in *Drosophila*, causing gut microbiota dysbiosis. To further explore the role of the gut microbiome in the observed gut phenotype and sleep, we used antibiotic cocktail treatment^42^, ^44^ to alter the gut microbiome in flies (Figure 5K–O). The microbiota had little effect on the ISC proliferation and sleep of flies in homeostatic conditions^60^ (Figures 5P,Q and S3E). Interestingly, we observed a decline in ISC proliferation (Figures 5P and S3E) and an increase in sleep (Figure 5Q) in *sss* mutants under antibiotic conditions. Therefore, we speculate that there exists a bidirectional interaction between gut microbiota and sleep loss in *sss* ^P1^/*sss* ^Δ40^ flies.

### 3.6 SSS in the brain regulates ISC proliferation through the GABA signalling pathway

**FIGURE 6:**
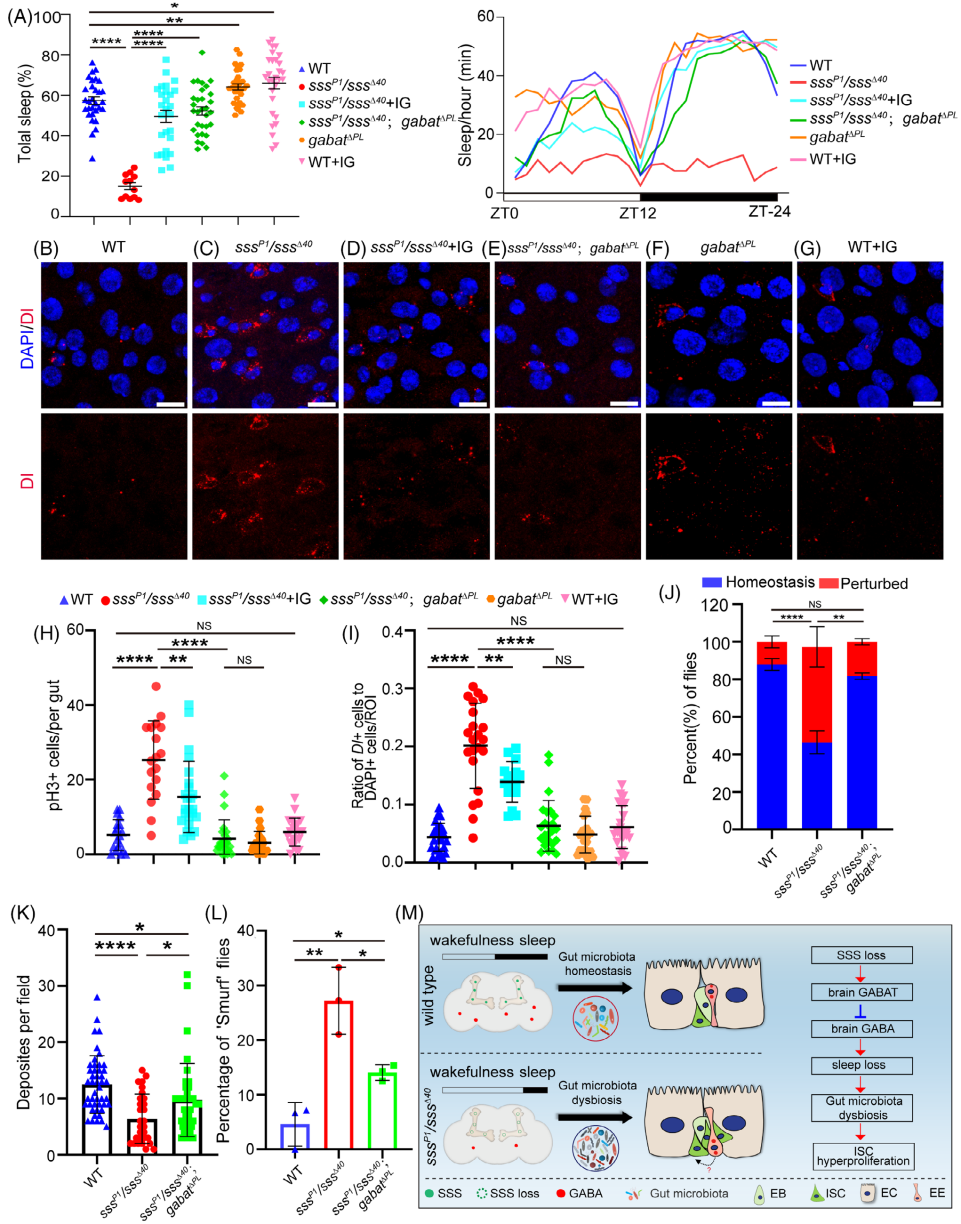
SSS regulates ISC proliferation partially through the GABA signalling pathway. (A) Total sleep (left) and sleep per hour (right) of the indicated genotypes. Mean and SEM. (B–G) Representative immunofluorescence images of Dl staining (red; ISC marker) in the R4 or R5 region of the midguts of the indicated genotypes. (H) Quantification of the number of pH3^+^ cells in the gut with the indicated genotype. Each dot corresponds to one gut. Mean and SD. (I) Quantification of Dl^+^ cell ratio per 3.4 × 10^4^ μm^2^ in the midgut with the indicated genotype. Each dot corresponds to one ROI. Mean and SD. (J) Quantification of the percentage of gut acid–base homeostasis in flies with the indicated genotypes. Fisher's exact test. **p < 0.01, ****p < 0.0001, NS = not significant. (K) Quantification of fly excretions from the above indicated genotypes. Mean and SD. (L) Quantification of the percentage of ‘Smurf’ flies in the indicated genotypes. Error bars represent the SD of three independent experiments. (M) A model illustrating the role of *sss* in controlling ISC proliferation and intestinal function through the brain‐to‐gut communication. Scale bar = 10 μm. DAPI‐stained nuclei are shown in blue. WT = wild type, Student's *t* test, *p < 0.05, **p < 0.01, ****p < 0.0001, NS = not significant.

Previous studies showed that *sss* mutants exhibit a decrease in gamma‐aminobutyric acid (GABA) in the brain due to the increased levels of GABA transaminase (GABAT, the enzyme breaking down GABA in glial cells).^37^ Therefore, the potential role of the GABA signalling pathway in the *sss* regulation of ISC proliferation and gut function was explored. GABAT mutation (*gabat* ^PL00338^) and IG (an agonist of the GABA_A_ receptor) were used to increase GABA levels in *sss* mutants for sleep recovery^36^ (Figure 6A). Both the depletion of *gabat* and feeding the flies with IG reduced pH3^+^ cells and Dl^+^ cells in the *sss* mutant flies (Figures 6B–I and S4A). The evaluation of the intestinal function also showed that the increase of GABA restored the intestinal barrier and digestive function in the *sss* mutants (Figure 6J–L). We further explore the potential role of GABA signalling in dysbiosis. While the CFU assay showed that modulating GABA signalling in *sss* mutants, the rescue of dysbiosis microbiota was some improvement, but not significant in flies with the administration of GABA agonists (Figure S4B–H). These data suggest GABA signalling has a limited influence on the gut microbiota in *sss* mutants. These above results suggested that SSS regulated ISC proliferation and intestinal function partially through gut microbiota and the GABA signalling pathway.

## 4 DISCUSSION

Over the past decade, studies on the effect of sleep loss on the intestine have mainly focused on immunity and inflammation.^18^ However, the sleep‐gut crosstalk is still largely unexplored due to the complexity of sleep. This study reveals that ISCs are involved in sleep‐regulating intestinal homeostasis and intestinal function in *Drosophila*. The *sss* mutants exhibiting obvious sleep loss caused abnormal activation of ISCs, impaired tissue regeneration, and disturbed intestinal function. Moreover, gut microbiota dysbiosis played a role in this process. The intestinal phenotype was also partially rescued by modulating the classical sleep‐promoting GABA pathway (Figure 6M).

There has been a lot of interest in figuring out how the brain communicates with the gut. According to recent research, the brain–gut axis is responsible for bidirectional communication between the brain and the intestine, which occurs through multiple pathways that include neural pathways, neurotransmitters, immune mediators, and gut microbiota.^61^, ^62^, ^63^ Our studies found that antibiotic treatment of *sss* ^P1^/*sss* ^Δ40^ flies (Figure 5M,N) resulted in a significant reduction in stem cell division (Figure 5P) and increased sleep time (Figure 5Q). This indicated that gut microbiota were involved in sleep‐regulating intestinal homeostasis. To more precisely detect target bacteria and their metabolites, additional investigations combining metagenomic analysis and metabolomic assays will be required in the future. Meanwhile, activation of the GABA pathway can rescue sleep behaviour (Figure 6A) and intestinal phenotype (Figure 6B–L). This finding suggested that the GABA pathway may also play a role in modulating sleep‐gut crosstalk. Nevertheless, research revealed that GABA and GABA receptors are also expressed in some EE cells in the gastrointestinal tract.^64^ Further studies are needed to determine whether intestinal GABA has a regulatory effect on ISC proliferation.

This study has some limitations due to the complexity of sleep regulation and sleep functions. Mechanical stimulation is a common method to induce acute sleep deprivation in *Drosophila*.^24^ However, great differences exist among individuals exposed to mechanical vibrations: one is sensitive to mechanical sleep deprivation and the other one is not, which led to the relatively large variation of gut proliferation between these flies after mechanical sleep deprivation (Figure 1B). And compared with *sss* mutant, mechanical sleep deprivation produced different results in the excretion experiment (Figures 1K,L and 3C). It was possible that the mechanical deprivation was only a short‐term acute sleep loss, in which case the impairment of some intestinal function was minimal. Our results demonstrate that *tub*‐*Gal4*‐driven *UAS*‐*sss* rescued the intestinal phenotype of *sss* mutants, while the rescue effects of *elav*‐*Gal4* (BL# 8760) were weaker. This was probably due to the low efficiency of *elav*‐*Gal4* or the extra expression of *sss* in addition to the neuronal cells driven by *elav*‐*Gal4*; thus, this aspect needs further exploration.

The precise ortholog of *sss* in mammals has not been found yet, and technical limitations could be the reason, at least in part: the coding region of the *sss* gene is too small to analyse. Intriguingly, lynx1 is considered as a mammalian homologue of SSS in some current studies.^65^ SSS and lynx1 have less than 20% amino acid identity, while lynx1 can perform sufficiently SSS‐like in vivo.^65^ However, SSS‐related Shaker channel and GABA pathways are conserved in mammals.^66^ In conclusion, the *sss* mutant is a model of sleep loss and worth investigating. Mouse models should be used in future studies to further verify their conservativeness and demonstrate whether sleep deprivation regulates mammalian ISCs.

In summary, our work highlights the effect of sleep loss on the role of ISCs in regulating tissue repair and intestinal function, providing a new perspective on the relationship between sleep and the intestine, and offering the possibility of the potential therapeutic intervention of intestinal disorders in patients with sleep deprivation via gut microbiota and the GABA pathway.

## AUTHOR CONTRIBUTIONS

Juanyu Zhou and Haiyang Chen initiated the project and designed the research; Juanyu Zhou, Mengyou Liu, Xiaoxin Guo, La Yan, Zehong Zhang, Gang Du and Zhendong Zhong performed the experiments, data collection, and analysis. Juanyu Zhou prepared figures under the supervision of Li He and Haiyang Chen. Juanyu Zhou and Mengyou Liu wrote the manuscript. Li He and Haiyang Chen proofread and gave advice. All authors read and approved the final manuscript.

## CONFLICT OF INTEREST STATEMENT

The authors declare no conflicts of interest.

## Supporting information


**Figure S1.** Sleep deprivation disturbs ISC proliferation and intestinal epithelial repair, related to Figure 1. (A and B) Total sleep (A) and rebound sleep (B) of sleep deprivation and non‐sleep deprivation flies. Mean and SEM. (C) Representative midgut pH3 staining images of sleep deprivation and non‐sleep deprivation flies. The yellow arrows indicate the pH3^+^ cells. (D) A schema of the injury‐and‐recovery experiment of sleep deprivation and non‐sleep deprivation flies. (E) Representative midgut pH3 staining images of injury‐and‐recovery experiment in sleep deprivation and non‐sleep deprivation flies. The yellow arrows indicate the pH3^+^ cells. (F–H) The interaction graphs related to Figure 1F–H. Mean and SEM. Scale bar = 100 μm. DAPI‐stained nuclei are shown in blue. Student's *t* test, *p < 0.05, **p < 0.01, ***p < 0.001, ****p < 0.0001, NS = not significant.

**Figure S2.** SSS regulates ISC proliferation and intestinal epithelial repair in *Drosophila*, related to Figures 2 and 3. (A) Representative Dl immunofluorescence images from the midgut R4 or R5 sections in the *Gal4* and *UAS* controls. Scale bar = 10 μm. (B) Representative midgut pH3 staining images with the indicated genotypes. The yellow arrows indicate the pH3^+^ cells. Scale bar = 100 μm. (C) Total sleep and sleep per hour of *actin* ^*ts*^ *> sss‐RNAi* and *actin* ^*ts*^ *> UAS‐lacZ*. After eclosion, flies were put at 18°C during the day and 29°C at night to deprive sleep only at night. Mean and SEM. (D) Quantification of the number of pH3^+^ cells in the gut with the indicated genotypes. Each dot corresponds to one gut. Mean and SD. (E) Representative midgut pH3 staining images of injury‐and‐recovery experiment in WT and *sss* mutant flies. The yellow arrows indicate the pH3^+^ cells. Scale bar = 100 μm. (F) Representative midgut pH3 staining images of ISC division rhythm in the 12:12 h LD cycle between ZT0 and ZT12 in CS and *sss* mutant flies with BLM. The yellow arrows indicate the pH3^+^ cells. Scale bar = 100 μm. (G) Quantification of the number of pH3^+^ cells in the gut of the indicated genotypes. Each dot corresponds to one gut. Mean and SD. (H) *sss* relative mRNA expression between sleep deprivation and non‐sleep deprivation flies. Mean and SD. (I) Quantification of the number of pH3^+^ cells in the gut of the indicated genotypes. Each dot corresponds to one gut. Mean and SD. (J) Representative DHE staining images of the midgut in WT and *sss* mutant flies. DHE staining shows the levels of ROS and Hochest‐stained nuclei are shown in blue. Scale bar = 100 μm. DAPI‐stained nuclei are shown in blue. Student's *t* tests, *p < 0.05, ****p < 0.0001, NS = not significant.

**Figure S3.** Disruption of SSS causes gut microbiota dysbiosis in *Drosophila*, related to Figure 5. (A–D) Heatmap of the normalized relative abundance of OTUs significantly changed between WT and *sss* ^P1^/*sss* ^Δ40^ flies at the phylum (A), family (B), genus (C), and species (D) level by one‐way ANOVA, *p* < 0.05. (E) Representative midgut pH3 staining images of WT and *sss* ^P1^/*sss* ^Δ40^ flies fed with and without antibiotics. The yellow arrows indicate the pH3^+^ cells. DAPI‐stained nuclei are shown in blue. Scale bar = 100 μm.

**Figure S4.** SSS regulates ISC proliferation partially through the GABA signalling pathway, related to Figure 6. (A) Representative midgut pH3 staining images of the indicated genotypes. The yellow arrows indicate the pH3^+^ cells. DAPI‐stained nuclei are shown in blue. Scale bar = 100 μm. (B–H) Representative images (B–G) and quantification (H) of the bacterial load in flies with the indicated genotypes. Mean and SD. Student's *t* tests, *p < 0.05, **p < 0.01, NS = not significant.

**Table S1.** Full *Drosophila* genotypes as they appear in each figure panel, related to Figures 1, 2, 3, 4, 5, 6 and [Link], [Link].

**Table S2.** (related to Materials and Methods). Reagent table

**Table S3.** List of changed gut microbiota between WT and sss^P1^/sss^Δ40^ flies by one‐way ANOVA, related to Figures 5 and S3.

## Data Availability

All 16S rRNA datasets are publicly available in Sequence Read Archive (SRA) BioProject: PRJNA874194.
