# Supplementary figures and images for: Sleep loss impairs intestinal stem cell function and gut homeostasis through the modulation of the GABA signalling pathway in Drosophila
Source: Cell Prolif. 2023 Mar 3;56(9):e13437. doi: 10.1111/cpr.13437 (PMC10472530; doi:10.1111/cpr.13437)

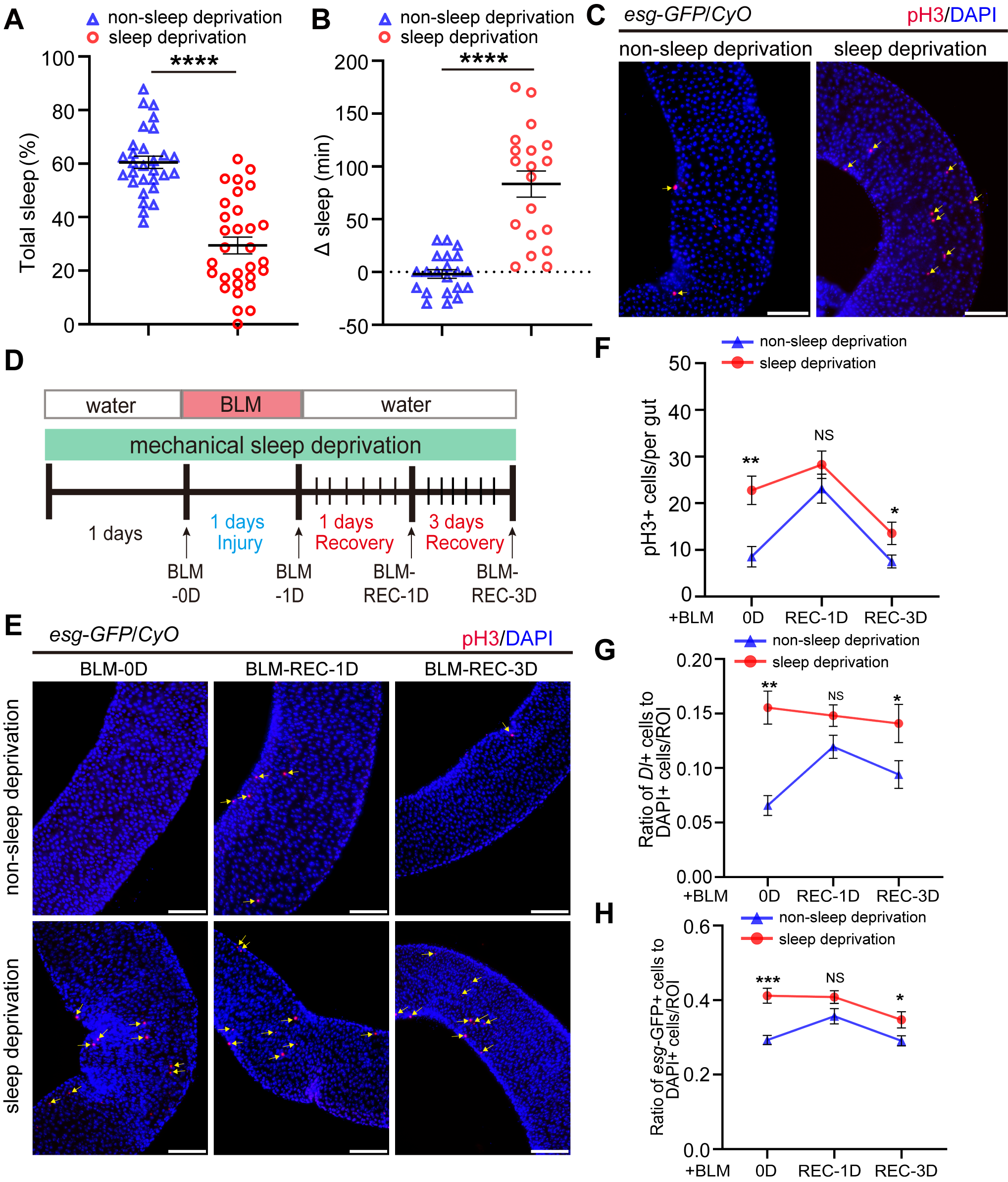

Supplement: Supplementary file 1 — Figure S1. Sleep deprivation disturbs ISC proliferation and intestinal epithelial repair, related to Figure 1. (A and B) Total sleep (A) and rebound sleep (B) of sleep deprivation and non‐sleep deprivation flies. Mean and SEM. (C) Representative midgut pH3 staining images of sleep deprivation and non‐sleep deprivation flies. The yellow arrows indicate the pH3+ cells. (D) A schema of the injury‐and‐recovery experiment of sleep deprivation and non‐sleep deprivation flies. (E) Representative midgut pH3 staining images of injury‐and‐recovery experiment in sleep deprivation and non‐sleep deprivation flies. The yellow arrows indicate the pH3+ cells. (F–H) The interaction graphs related to Figure 1F–H. Mean and SEM. Scale bar = 100 μm. DAPI‐stained nuclei are shown in blue. Student's t test, *p < 0.05, **p < 0.01, ***p < 0.001, ****p < 0.0001, NS = not significant. [file CPR-56-e13437-s006.tif]

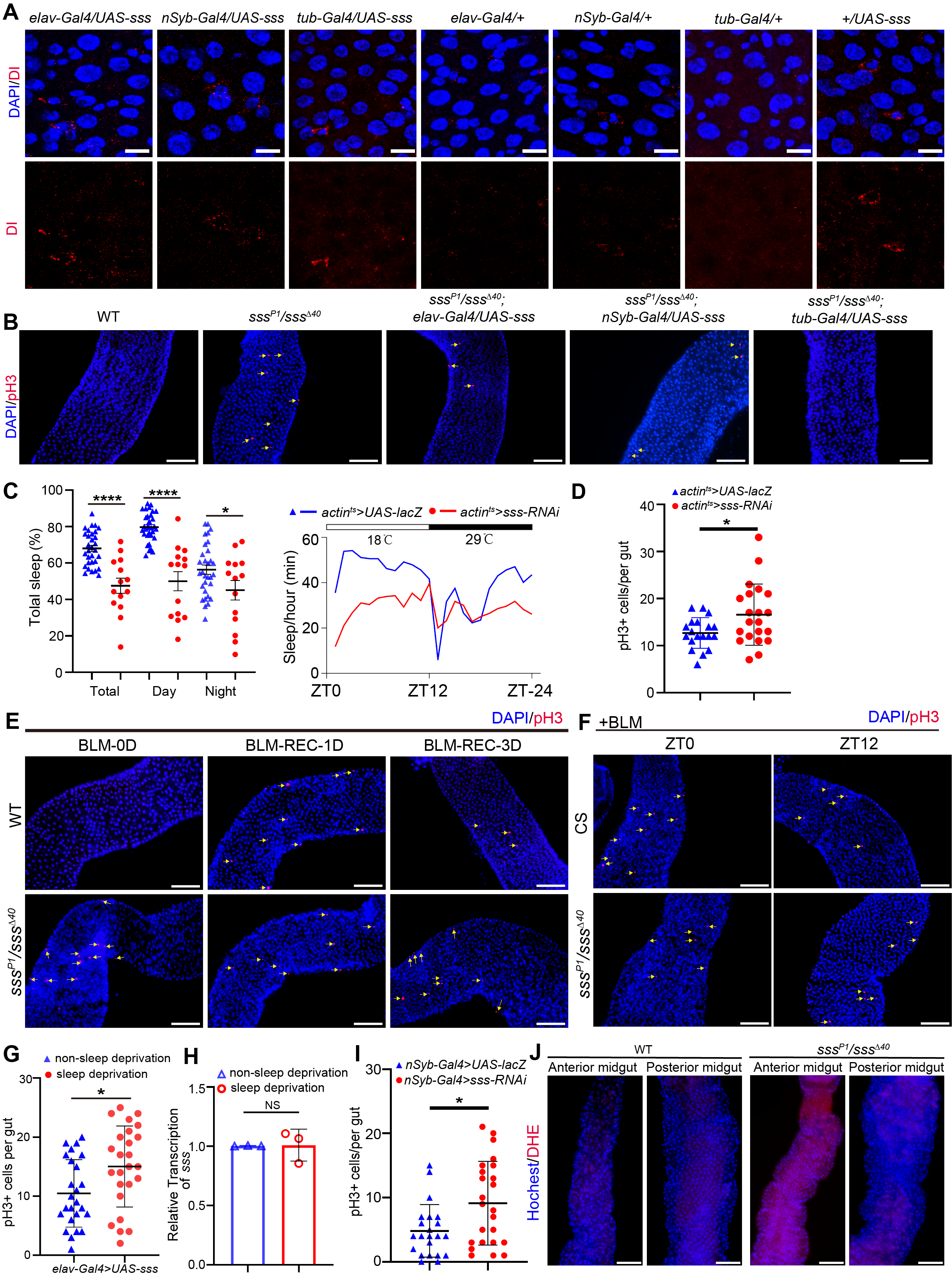

Supplement: Supplementary file 2 — Figure S2. SSS regulates ISC proliferation and intestinal epithelial repair in Drosophila, related to Figures 2 and 3. (A) Representative Dl immunofluorescence images from the midgut R4 or R5 sections in the Gal4 and UAS controls. Scale bar = 10 μm. (B) Representative midgut pH3 staining images with the indicated genotypes. The yellow arrows indicate the pH3+ cells. Scale bar = 100 μm. (C) Total sleep and sleep per hour of actin ts > sss‐RNAi and actin ts > UAS‐lacZ. After eclosion, flies were put at 18°C during the day and 29°C at night to deprive sleep only at night. Mean and SEM. (D) Quantification of the number of pH3+ cells in the gut with the indicated genotypes. Each dot corresponds to one gut. Mean and SD. (E) Representative midgut pH3 staining images of injury‐and‐recovery experiment in WT and sss mutant flies. The yellow arrows indicate the pH3+ cells. Scale bar = 100 μm. (F) Representative midgut pH3 staining images of ISC division rhythm in the 12:12 h LD cycle between ZT0 and ZT12 in CS and sss mutant flies with BLM. The yellow arrows indicate the pH3+ cells. Scale bar = 100 μm. (G) Quantification of the number of pH3+ cells in the gut of the indicated genotypes. Each dot corresponds to one gut. Mean and SD. (H) sss relative mRNA expression between sleep deprivation and non‐sleep deprivation flies. Mean and SD. (I) Quantification of the number of pH3+ cells in the gut of the indicated genotypes. Each dot corresponds to one gut. Mean and SD. (J) Representative DHE staining images of the midgut in WT and sss mutant flies. DHE staining shows the levels of ROS and Hochest‐stained nuclei are shown in blue. Scale bar = 100 μm. DAPI‐stained nuclei are shown in blue. Student's t tests, *p < 0.05, ****p < 0.0001, NS = not significant. [file CPR-56-e13437-s005.tif]

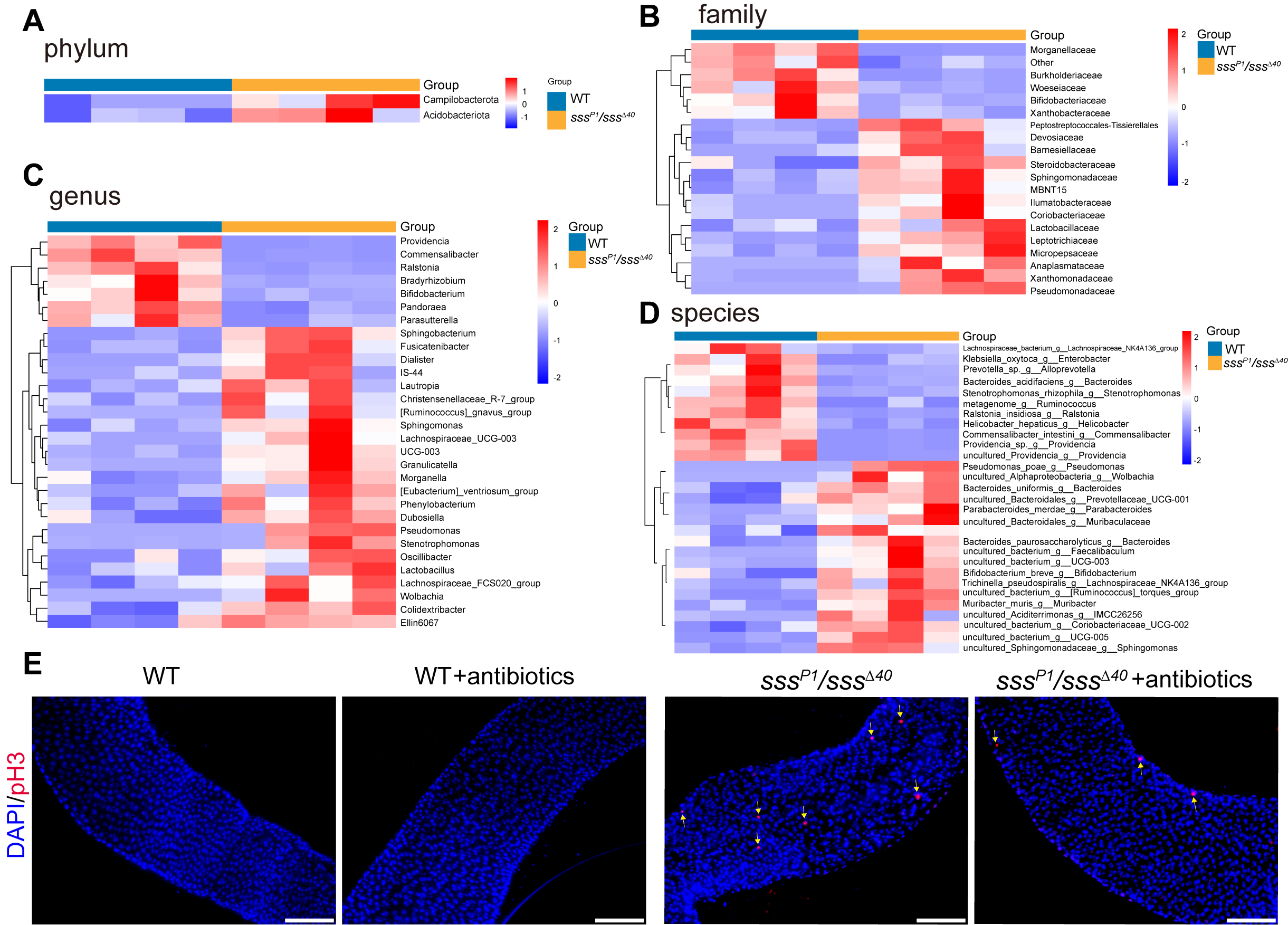

Supplement: Supplementary file 3 — Figure S3. Disruption of SSS causes gut microbiota dysbiosis in Drosophila, related to Figure 5. (A–D) Heatmap of the normalized relative abundance of OTUs significantly changed between WT and sss P1/sss Δ40 flies at the phylum (A), family (B), genus (C), and species (D) level by one‐way ANOVA, p < 0.05. (E) Representative midgut pH3 staining images of WT and sss P1/sss Δ40 flies fed with and without antibiotics. The yellow arrows indicate the pH3+ cells. DAPI‐stained nuclei are shown in blue. Scale bar = 100 μm. [file CPR-56-e13437-s004.tif]

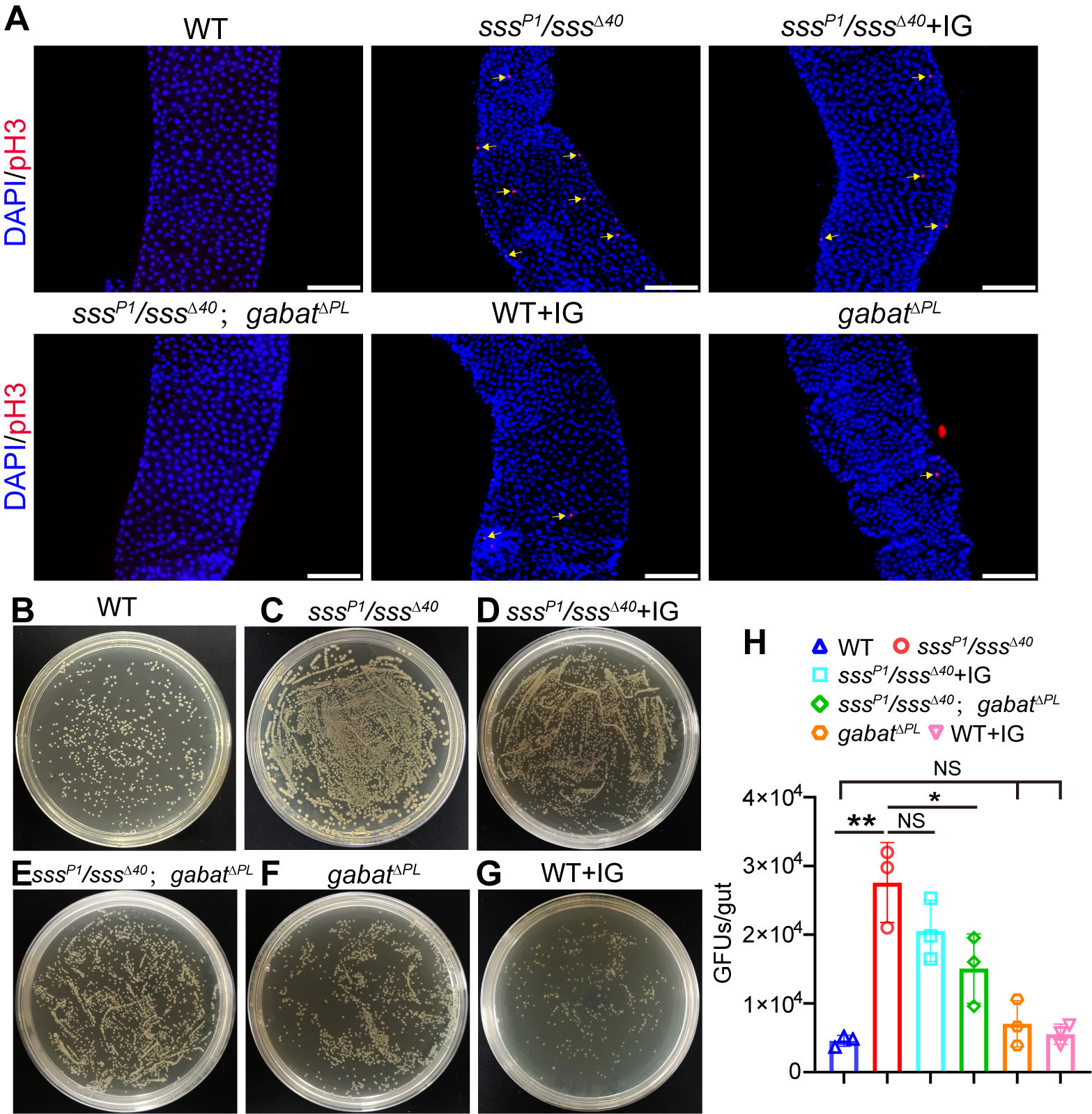

Supplement: Supplementary file 4 — Figure S4. SSS regulates ISC proliferation partially through the GABA signalling pathway, related to Figure 6. (A) Representative midgut pH3 staining images of the indicated genotypes. The yellow arrows indicate the pH3+ cells. DAPI‐stained nuclei are shown in blue. Scale bar = 100 μm. (B–H) Representative images (B–G) and quantification (H) of the bacterial load in flies with the indicated genotypes. Mean and SD. Student's t tests, *p < 0.05, **p < 0.01, NS = not significant. [file CPR-56-e13437-s001.tif]
