# Supplementary material for: Sleep loss impairs intestinal stem cell function and gut homeostasis through the modulation of the GABA signalling pathway in Drosophila
Source: Cell Prolif. 2023 Mar 3;56(9):e13437. doi: 10.1111/cpr.13437 (PMC10472530; doi:10.1111/cpr.13437)
Supplement: Supplementary file 5 — Table S1. Full Drosophila genotypes as they appear in each figure panel, related to Figures 1, 2, 3, 4, 5, 6 and [Link], [Link]. [file CPR-56-e13437-s002.docx]

| **Supplementary Table S1. Full *Drosophila* genotypes as they appear in each figure panel, related to Figure 1-6 and S1-S4.** | |
| --- | --- |
| **Figure 1** | |
| **A, E, I, K** | *w^-^; esg-GFP/CyO; +/+* |
| **Figure 2** | |
| **C** | *w^-^; + /+; +/+* |
| **D** | *w^-^; sss^P1^/sss^Δ40^; +/+* |
| **E** | *w^-^; sss^P1^/sss^Δ40^; elav-Gal4/UAS-sss* |
| **F** | *w^-^; sss^P1^/sss^Δ40^; nSyb-Gal4/UAS-sss* |
| **G** | *w^-^; sss^P1^/sss^Δ40^; tub-Gal4/UAS-sss* |
| **J** | *w^-^; + /+; +/+* |
|  | *w^-^; sss^P1^/sss^Δ40^; +/+* |
| **M, N** | Canton-S |
|  | *w^-^; sss^P1^/sss^Δ40^; +/+* |
| **Figure 3** | |
| **A, C, D, E** | *w^-^; + /+; +/+* |
|  | *w^-^; sss^P1^/sss^Δ40^; +/+* |
| **Figure 4** | |
| **B, C, E, F, G** | *w^-^; sss-GFP/ CyO; +/+* |
| **Figure 5** | |
| **A, K, L** | *w^-^; + /+; +/+* |
| **B, M, N** | *w^-^; sss^P1^/sss^Δ40^; +/+* |
| **C** | *w^-^; sss^P1^/sss^Δ40^; elav-Gal4/UAS-sss* |
| **D** | *w^-^; sss^P1^/sss^Δ40^; tub-Gal4/UAS-sss* |
| **Figure 6** | |
| **B, G** | *w^-^; + /+; +/+* |
| **C, D** | *w^-^; sss^P1^/sss^Δ40^; +/+* |
| **E** | *w^-^; sss^P1^/sss^Δ40^; gabat^PL^ /gabat^PL^* |
| **F** | *w^-^; + /+; gabat^PL^ /gabat^PL^* |
| **Figure S1** | |
| **C, E** | *w^-^; esg-GFP/CyO; +/+* |
| **Figure S2** | |
| **A** | *w^-^; + /+; elav-Gal4/UAS-sss* |
|  | *w^-^; + /+; nSyb-Gal4/UAS-sss* |
|  | *w^-^; + /+; tub-Gal4/UAS-sss* |
|  | *w^-^; + /+; elav-Gal4/+* |
|  | *w^-^; + /+; nSyb-Gal4/+* |
|  | *w^-^; + /+; tub-Gal4/+* |
|  | *w^-^; + /+; UAS-sss /+* |
| **B** | *w^-^; + /+; +/+* |
|  | *w^-^; sss^P1^/sss^Δ40^; +/+* |
|  | *w^-^; sss^P1^/sss^Δ40^; elav-Gal4/UAS-sss* |
|  | *w^-^; sss^P1^/sss^Δ40^; nSyb-Gal4/UAS-sss* |
|  | *w^-^; sss^P1^/sss^Δ40^; tub-Gal4/UAS-sss* |
| **C** | *w^-^; tub80^ts^/UAS-lacZ; actin-Gal4/+* |
|  | *w^-^; tub80^ts^/UAS-sss-RNAi; actin-Gal4/+* |
| **E, J** | *w^-^; + /+; +/+* |
|  | *w^-^; sss^P1^/sss^Δ40^; +/+* |
| **F** | Canton-S |
|  | *w^-^; sss^P1^/sss^Δ40^; +/+* |
| **G** | *w^-^; + /+; elav-Gal4/ UAS-sss* |
| **H** | *w^-^; esg-GFP/CyO; +/+* |
| **I** | *w^-^; UAS-lacZ/+; nSyb-Gal4/+* |
|  | *w^-^; UAS-sss-RNAi /+; nSyb-Gal4/+* |
| **Figure S3** |  |
| **E** | *w^-^; + /+; +/+* |
|  | *w^-^; sss^P1^/sss^Δ40^; +/+* |
| **Figure S4** |  |
| **B, G** | *w^-^; + /+; +/+* |
| **C, D** | *w^-^; sss^P1^/sss^Δ40^; +/+* |
| **E** | *w^-^; sss^P1^/sss^Δ40^; gabat^PL^ /gabat^PL^* |
| **F** | *w^-^; + /+; gabat^PL^ /gabat^PL^* |
