# Supplementary material for: Sleep loss impairs intestinal stem cell function and gut homeostasis through the modulation of the GABA signalling pathway in Drosophila
Source: Cell Prolif. 2023 Mar 3;56(9):e13437. doi: 10.1111/cpr.13437 (PMC10472530; doi:10.1111/cpr.13437)
Supplement: Supplementary file 6 — Table S2. (related to Materials and Methods). Reagent table [file CPR-56-e13437-s007.docx]

| **Supplementary Table S2 (related to Materials and Methods). Reagent Table** | | |
| --- | --- | --- |
| **Reagent** | **Source** | **Dilution** |
| Chicken polyclonal anti-GFP | Abcam Cat# ab13970 RRID:AB_300798 | 1: 1000 |
| Mouse anti-Delta | DSHB Cat# C594.9B RRID:AB_528194 | 1: 100 |
| Mouse anti-Prospero | DSHB Cat# MR1A RRID:AB_528440 | 1: 200 |
| Rabbit anti-phosphoHistone H3 (Ser10) | Millipore Cat# 06-570 RRID:AB_310177 | 1: 1000 |
